# Supplementary material for: Multi‐dataset identification of innovative feature genes and molecular mechanisms in keratoconus
Source: J Cell Mol Med. 2024 Sep 19;28(18):e70079. doi: 10.1111/jcmm.70079 (PMC11412914; doi:10.1111/jcmm.70079)
Supplement: Supplementary file 2 — Material S2. [file JCMM-28-e70079-s002.zip › 7.GSEA analysis/5.RNF217-AS1/KEGG_LYSINE_DEGRADATION.html]

Details for gene set KEGG\_LYSINE\_DEGRADATION[GSEA]

|  || Dataset | RNF217-AS1.group.cls#h\_versus\_l |
| Phenotype | group.cls#h\_versus\_l |
| Upregulated in class | l |
| GeneSet | KEGG\_LYSINE\_DEGRADATION |
| Enrichment Score (ES) | -0.46980694 |
| Normalized Enrichment Score (NES) | -1.5101964 |
| Nominal p-value | 0.057377048 |
| FDR q-value | 0.3194242 |
| FWER p-Value | 0.746 |
Table: GSEA Results Summary

  

Fig 1: Enrichment plot: KEGG\_LYSINE\_DEGRADATION      
 Profile of the Running ES Score & Positions of GeneSet Members on the Rank Ordered List

  

| SYMBOL | TITLE | RANK IN GENE LIST | RANK METRIC SCORE | RUNNING ES | CORE ENRICHMENT || 1 | NSD1 | na | 7789 | 0.133 | -0.1926 | No |
| 2 | DLST | na | 8592 | 0.114 | -0.1882 | No |
| 3 | ASH1L | na | 9684 | 0.092 | -0.1975 | No |
| 4 | TMLHE | na | 12340 | 0.051 | -0.2618 | No |
| 5 | DOT1L | na | 12510 | 0.049 | -0.2550 | No |
| 6 | OGDHL | na | 12934 | 0.043 | -0.2569 | No |
| 7 | BBOX1 | na | 13063 | 0.041 | -0.2506 | No |
| 8 | PIPOX | na | 13470 | 0.037 | -0.2535 | No |
| 9 | AASDH | na | 16274 | 0.006 | -0.3328 | No |
| 10 | SETDB2 | na | 16474 | 0.004 | -0.3376 | No |
| 11 | ACAT2 | na | 17362 | -0.005 | -0.3619 | No |
| 12 | OGDH | na | 17698 | -0.008 | -0.3697 | No |
| 13 | EHMT1 | na | 19331 | -0.024 | -0.4110 | No |
| 14 | ALDH3A2 | na | 20121 | -0.031 | -0.4263 | No |
| 15 | HADHA | na | 20646 | -0.036 | -0.4327 | No |
| 16 | ECHS1 | na | 20997 | -0.040 | -0.4331 | No |
| 17 | SETDB1 | na | 21657 | -0.047 | -0.4408 | No |
| 18 | SETD1A | na | 22113 | -0.052 | -0.4414 | No |
| 19 | SETD2 | na | 22512 | -0.056 | -0.4393 | No |
| 20 | ACAT1 | na | 22576 | -0.057 | -0.4274 | No |
| 21 | ALDH9A1 | na | 23254 | -0.065 | -0.4314 | No |
| 22 | AASDHPPT | na | 24347 | -0.080 | -0.4435 | No |
| 23 | ALDH7A1 | na | 25259 | -0.095 | -0.4470 | Yes |
| 24 | PLOD3 | na | 25794 | -0.103 | -0.4376 | Yes |
| 25 | EHMT2 | na | 25927 | -0.106 | -0.4160 | Yes |
| 26 | HADH | na | 26099 | -0.109 | -0.3947 | Yes |
| 27 | SETD1B | na | 26690 | -0.120 | -0.3829 | Yes |
| 28 | SETMAR | na | 27289 | -0.132 | -0.3684 | Yes |
| 29 | EHHADH | na | 27336 | -0.133 | -0.3378 | Yes |
| 30 | PLOD2 | na | 28070 | -0.150 | -0.3227 | Yes |
| 31 | GCDH | na | 28300 | -0.156 | -0.2917 | Yes |
| 32 | ALDH1B1 | na | 28642 | -0.166 | -0.2616 | Yes |
| 33 | SUV39H2 | na | 28987 | -0.176 | -0.2292 | Yes |
| 34 | SETD7 | na | 29067 | -0.178 | -0.1886 | Yes |
| 35 | PLOD1 | na | 29560 | -0.193 | -0.1564 | Yes |
| 36 | AADAT | na | 31032 | -0.243 | -0.1402 | Yes |
| 37 | SUV39H1 | na | 32158 | -0.301 | -0.1003 | Yes |
| 38 | AASS | na | 32171 | -0.301 | -0.0282 | Yes |
| 39 | ALDH2 | na | 33631 | -0.423 | 0.0316 | Yes |
Table: GSEA details [plain text format]

  

Fig 2: KEGG\_LYSINE\_DEGRADATION      
 Blue-Pink O' Gram in the Space of the Analyzed GeneSet

  

Fig 3: KEGG\_LYSINE\_DEGRADATION: Random ES distribution      
 Gene set null distribution of ES for **KEGG\_LYSINE\_DEGRADATION**
